# Recurrent cutaneous collagenous vasculopathy in a heterozygote carrier for the ataxia telangiectasia mutated gene: A case report

**DOI:** 10.1016/j.jdcr.2025.05.019

**Published:** 2025-06-16

**Authors:** Chioma Alaka, Luke Tomczak, Erum N. Ilyas

**Affiliations:** a Drexel University College of Medicine, Philadelphia, Pennsylvania; b Department of Dermatology, Drexel University College of Medicine, Philadelphia, Pennsylvania

**Keywords:** ATM gene, CCV, cutaneous collagenous vasculopathy, telangiectasias, telangiectatic disorders

## Introduction

Cutaneous collagenous vasculopathy (CCV) is a rare microangiopathy of unclear etiology clinically manifesting as spreading telangiectasias predominantly involving the extremities.^1^ We report a unique case of a patient who is a carrier of the ataxia telangiectasia mutated (ATM) gene mutation with recurrent episodes of CCV separated by a decade with identifiable medication triggers.

## Case report

**Fig 1:**
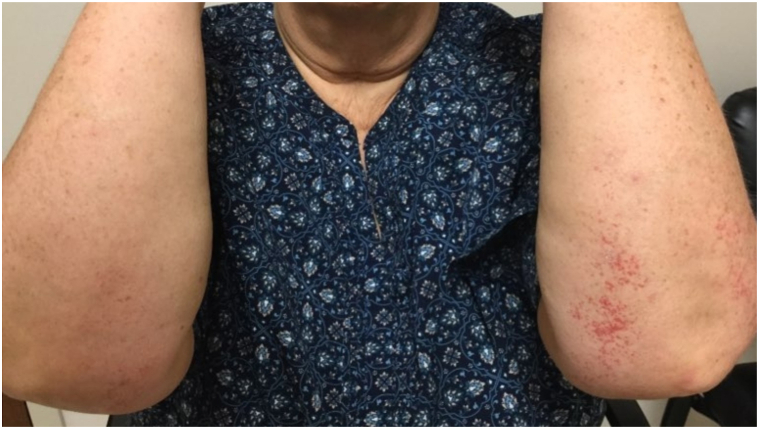
Cutaneous collagenous vasculopathy (CCV). First episode of CCV with isolated telangiectatic patches limited to extensor forearms distal to elbow.

**Fig 2:**
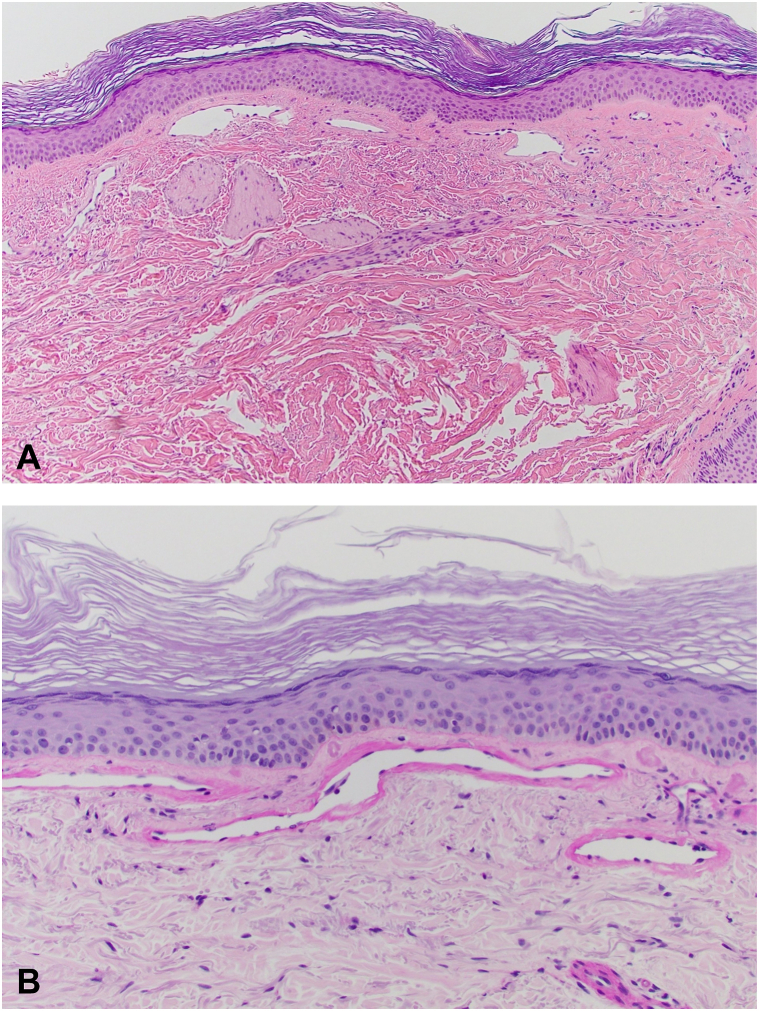
Cutaneous collagenous vasculopathy (CCV) histology noted on initial presentation. Please note that identical findings were seen on repeat biopsy at subsequent presentation. **A,** Hematoxylin and eosin–stained slide showing dilated vascular channels in the superficial dermis (original magnification: ×100). **B,** Dilated vascular channels showing amorphous PAS positive material within markedly thickened walls (original magnification: ×200). Photomicrographs courtesy of Igor Lomazoff, MD. *PAS*, Periodic acid-Schiff.

A 56-year-old Caucasian female initially presented to her primary care physician with the sudden onset of asymptomatic red patches limited to her extensor forearms while taking a short course of clarithromycin. Her medical history included hypertension and type 2 diabetes mellitus for which she was managed with labetalol and metformin. She was advised at the time that her skin changes were likely secondary to an “allergic reaction” to clarithromycin and instructed to discontinue the medication. These patches persisted for 3 years with no further worsening or spreading prior to seeking dermatologic evaluation. Physical examination revealed distinct blanching telangiectatic patches limited to the forearms distal to the elbows (Fig 1). Punch biopsy demonstrated dilated vascular channels showing amorphous Periodic acid-Schiff positive material within markedly thickened walls leading to a diagnosis of CCV (Fig 2, *A* and *B*). Treatment was successfully performed with pulsed dye laser to the limited areas of involvement. Routine skin cancer screenings performed annually in the office did not reveal recurrences or new patches.

Seven years later, at 66 years of age, she presented with new onset spreading red patches on her distal forearms, legs, and thighs over the course of 6 months. There was no oral or ocular involvement and no reported cutaneous or systemic symptoms other than exacerbation by heat, warmth, and showers. Her medical history at this point included hypertension and type 2 diabetes mellitus in addition to melanoma in situ treated by wide excision 6 years prior and recent conjunctival squamous cell carcinoma in situ of the left eye treated by surgical resection and 2 cycles of 5-fluorouracil drops. Genetic testing for cancer risk assessment was performed 4 years prior to her current presentation given her personal history of melanoma and family history of colon and uterine cancer. She was found to be a heterozygote carrier for a pathogenic mutation of the ATM gene called c.5763-1050A>G. Her long-term medications included labetalol, spironolactone, metformin, gabapentin, azelastine nasal spray, and cetirizine. Notably, she had been started on hydrochlorothiazide prior to the onset of her rash and was discontinued due to dehydration and leg cramps just prior to her dermatologic evaluation.

**Fig 3:**
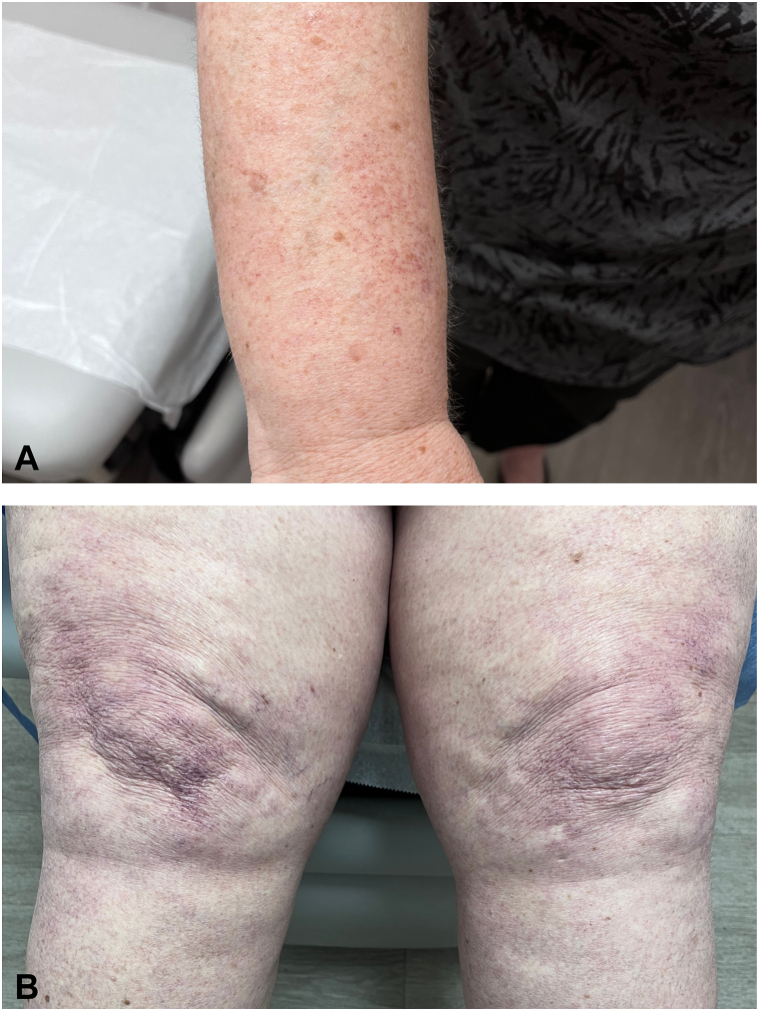
Cutaneous collagenous vasculopathy (CCV). **A,** Patches of telangiectasias over the right arm. **B,** Patches of telangiectasias over the legs bilaterally.

Physical examination revealed diffuse blanching telangiectatic patches extensively involving the arms, thighs, calves, and feet (Fig 3, *A* and *B*). Punch biopsy again demonstrated dilated superficial vascular channels with Periodic acid-Schiff stain showing thickening of vascular walls supporting a diagnosis of CCV identical to those noted on previous pathology (Fig 2, *A* and *B*). She noted that the progression of telangiectatic patches ceased after discontinuation of hydrochlorothiazide. Her cutaneous findings have been stable with no evidence of worsening or regression. No further treatment was pursued given the extensive involvement.

Of note, the patient’s sibling also underwent genetic testing as a part of a familial cancer risk assessment and was found to neither carry the same mutation nor share similar cutaneous findings.

## Discussion

CCV is a rare microangiopathy of unknown etiology with less than 100 cases reported in the literature but thought to be underdiagnosed.^1^ The condition predominantly affects older adults with pediatric cases identified and no significant gender predilection.^1^ Although many cases carry associated comorbid diagnoses such as hypertension, dyslipidemia, and type II diabetes mellitus and oral medications such as statins and angiotensin-converting enzyme inhibitors seen with notable consistency, it is unclear if these correlations are coincidental.^1^^,^^2^ Most often, telangiectasias develop on the lower extremities and spread upwards, while other reported patterns of telangiectatic spread have been reported.

Diagnosis of CCV is suggested by clinical picture related to age of onset distinguishing it from childhood telangiectatic syndromes such as hereditary benign telangiectasia, hereditary hemorrhagic telangiectasia (Osler-Weber-Rendu syndrome), and ataxia telangiectasia (Louis-Bar syndrome).^2^ Histologic findings of dilated superficial dermal blood vessels with thickened walls due to perivascular collagen deposition differentiate CCV from other causes of generalized telangiectasia such as generalized essential telangiectasia and telangiectatic mastocytosis.^2^^,^^3^

Given the limited knowledge of CCV based on case reports and case series found in the literature, the etiology is unclear. CCV has largely been distinguished from pediatric telangiectatic disorders based on clinical picture of age of onset and patterns of telangiectatic distribution. A genetic cancer risk assessment in this case revealed her to be a heterozygous carrier of a pathogenic mutation of the ATM gene called c.5763-1050A>G. There have been no prior reported cases of the ATM gene in CCV patients; however, the ATM gene is also associated with ataxia telangiectasia (Louis-Bar syndrome), a pediatric telangiectatic disorder.

Classic presentation of ataxia-telangiectasia syndrome presents as clinically distinct from CCV with pediatric age of onset, ataxia presenting in childhood, and telangiectasias favoring ocular and auricle involvement.^4^ Among several roles for the ATM gene to play in the regulation of the cell cycle linking it to various clinical manifestations, its role in DNA repair and angiogenesis link it to cancer and telangiectasia formation, respectively.^5^^,^^6^ There have been no reported distinct cutaneous findings noted in heterozygous ATM gene carriers; however, an association with diabetes and cancer (breast, in particular) has been noted.^7^ There are 2 reports of histologic findings associated with telangiectasias seen in ataxia telangiectasia. One demonstrated dilated venules with no comment made on vascular wall thickening,^8^ while another showed evidence of hyalinization of blood vessels as one of several vascular changes noted in the brain parenchyma.^9^ Early reports suggested ataxia telangiectasia as linked to collagen disorders given pathologic findings on autopsy of extensive collagen deposition in the skin.^10^ Interestingly the first report of CCV suggested the possibility of histologic findings as related to disorganized collagen production in response to vascular repair.^3^

To our knowledge, this is the first reported case identifying a known ATM gene carrier with recurrent CCV. Given the known role of the ATM gene in angiogenesis, a possible etiologic connection may be possible as well as a role for triggering agents given its role in regulation of cell cycles.

## Conclusion

This case underscores the possibility of CCV to serve as a cutaneous manifestation of heterozygous ATM gene carriers, predisposing individuals toward its development in response to triggering agents. Identifying this relation may offer further support for consideration to genetic testing to identify ATM gene carriers based on cutaneous findings given its association with familial cancer predisposition.

## Conflicts of interest

None disclosed.
